# *In situ* study of sigma phase formation in Cr–Co–Ni ternary alloys at 800°C using the long duration experiment facility at Diamond Light Source

**DOI:** 10.1107/S1600577518009475

**Published:** 2018-08-13

**Authors:** L. D. Connor, P. M. Mignanelli, K. A. Christofidou, N. G. Jones, A. R. Baker, C. C. Tang, S. Guérin, H. J. Stone

**Affiliations:** a Diamond Light Source Limited, Diamond House, Harwell Science and Innovation Campus, Didcot, Oxfordshire OX11 0DE, UK; b Department of Materials Science and Metallurgy, University of Cambridge, Cambridge CB3 0FS, UK; c Ilika technologies Ltd, Kenneth Dibben House, Enterprise Road, Southampton SO16 7NS, UK

**Keywords:** sigma, TCP (topologically close-packed phase), synchrotron, X-ray powder diffraction, SEM (scanning electron microscopy), ternary alloys, metallurgial phenomena

## Abstract

The new long duration experiment facility on beamline I11 at Diamond Light Source has been used to study the kinetics of sigma phase formation in three Cr–Co–Ni alloys. Diffraction data acquired during *in situ* exposure at 800°C for 50 d showed progressive increases in the sigma fraction. This was accompanied by changes in the proportions of the other phases, which differed markedly between the alloys studied. These results demonstrate the capabilities of the long duration facility for the study of metallurgical phenomena over periods of months to years, a capability not previously available at a synchrotron source.

## Abstract 2

The capabilities of the new long duration experiment facility at Diamond Light Source are demonstrated through a study of phase evolution in Cr–Co–Ni alloys using *in situ* X-ray diffraction.

## 1. Introduction   

Many commercially important alloys exhibit microstructural and phase changes that occur over time scales that may extend from months to years. These include topologically close-packed phase formation in the nickel-base superalloys used in gas turbine engines (Simonetti & Caron, 1998 ▸; Tin & Pollock, 2003 ▸; Acharya & Fuchs, 2004 ▸; Yang *et al.*, 2007 ▸), phase separation and intermetallic precipitation in iron-based alloys used in power generation (Chung & Leax, 1990 ▸; Murayama *et al.*, 1999 ▸; Danoix & Auger, 2000 ▸; Sourmail, 2001 ▸), and microstructural evolution in aluminium alloys (Braun, 2006 ▸; Katsikis *et al.*, 2008 ▸; Lukina *et al.*, 2011 ▸). Traditionally, these phenomena have been studied by *ex situ* examination of pre-exposed samples. Whilst often effective, this approach may be compromised by additional changes that occur on removing the samples from the exposure environment and the effect of sample-to-sample variations. The newly commissioned Long Duration Experiment (LDE) facility (Murray *et al.*, 2017 ▸) on beamline I11 (Thompson *et al.*, 2009 ▸) at the Diamond Light Source synchrotron X-ray centre provides a unique facility for the periodic *in situ* acquisition of two-dimensional diffraction data for durations extending to years. To demonstrate the efficacy of this facility a study has been performed of sigma phase formation in a series of model Cr–Co–Ni alloys.

The tetragonal sigma phase is known to form in many commercial alloy systems, including stainless steels (Villanueva *et al.*, 2006 ▸; Sieurin & Sandström, 2007 ▸; Schwind *et al.*, 2000 ▸; Minami *et al.*, 1986 ▸) and nickel-base superalloys (Rae & Reed, 2001 ▸; Sato *et al.*, 2006 ▸; Wilson, 2017*a* ▸). In addition, the emerging field of high-entropy alloys has recently identified sigma precipitates across a range of multicomponent systems (Jones *et al.*, 2016 ▸; Pickering *et al.*, 2016 ▸). The occurrence of these phases is associated with deterioration in the mechanical properties, most notably the creep rupture life (Dreshfield & Ashbrook, 1969 ▸; Jones *et al.*, 2014 ▸). This reduction in properties is thought to be a consequence of the brittle sigma phase, offering sites for crack initiation and the depletion of solution strengthening refractory elements in the matrix (Sims *et al.*, 1987 ▸). The crystal structure of the tetragonal sigma phase (space group *P*4_2_/*mnm*) comprises 30 atomic sites (Yakel, 1983*a* ▸,*b* ▸). The flexibility of this structure to accommodate elements of differing atomic sizes permits it to exist across a wide range of stoichiometries in transition metal alloys. Importantly, in many systems the sigma phase is not congruently formed and instead precipitates through a solid-state reaction, which is often sluggish (Mitchell *et al.*, 2005 ▸).

As with other systems exhibiting sluggish phase transformations, experimental studies of sigma phase formation have typically relied upon *ex situ* examination of samples subjected to thermal exposures of varying duration. However, such studies may be compromised by inconsistencies in the microstructures of the samples due to differences in the initial condition following alloy processing and changes that occur during cooling from the thermal exposure. Furthermore, the low sigma fraction formed in commercially relevant alloys often prohibits the reliable characterization of this phase using laboratory methods on bulk samples. This typically necessitates the study of electrolytically extracted residues, although the accuracy of quantitative assessments using this method has not yet been established (Wilson, 2017*b* ▸). Synchrotron X-ray diffraction offers a method by which quantitative data may be acquired from a single bulk sample *in situ,* addressing many of the issues encountered with the use of multiple samples. However, conventional access routes to such facilities typically limit experimental durations to less than one week, periods that may well be insufficient to monitor sigma formation *in situ*.

Therefore, to gain insight into sigma phase formation and demonstrate the capability of the new LDE instrument, three model alloys from the Cr–Co–Ni system have been studied *in situ* at elevated temperature. These alloys were selected as they were expected to form appreciable volume fractions of the sigma phase. This enabled the reliable acquisition of temporally resolved X-ray diffraction data, providing detailed information on the crystallographic changes that occur from a single sample. In addition, these model alloys also allowed investigation of compositional space where recent results have suggested that two distinct sigma phases may exist (Connor *et al.*, 2016 ▸), in contrast to previous reports on this ternary system (Kaufman & Nesor, 1974 ▸). Analysis of the two-dimensional diffraction data acquired from individual alloys demonstrated the efficacy of the LDE facility, showing the kinetics of sigma precipitation, the evolution of metastable phases and how their associated crystal structures vary as a function of time.

## 2. Materials and methods   

Three model alloys were studied from the Cr–Co–Ni system, with nominal compositions of 50Cr–20Co–30Ni, 50Cr–25Co–25Ni and 50Cr–30Co–20Ni, which were expected to lie between the gamma (A1, Strukturbericht notation) and sigma (D8_b_, Strukturbericht notation) phase fields, of the Cr–Co–Ni ternary system (Kaufman & Nesor, 1974 ▸). The alloys were prepared as ∼60 g ingots by vacuum arc melting using elements of 99.9% purity or greater. To enhance chemical homogeneity, the ingots were inverted and remelted five times, prior to encapsulation in quartz ampoules under an argon atmosphere and solution heat treated for 4 h at 1250°C. To reduce the grain size to a level appropriate for powder diffraction, the homogenized ingots were sectioned and cold rolled, with a thickness reduction of −40%, before an annealing heat treatment of 1 h at 800°C. Samples approximately 8 mm in diameter and 0.2 mm thick were removed from the annealed material for the study.

**Figure 1:**
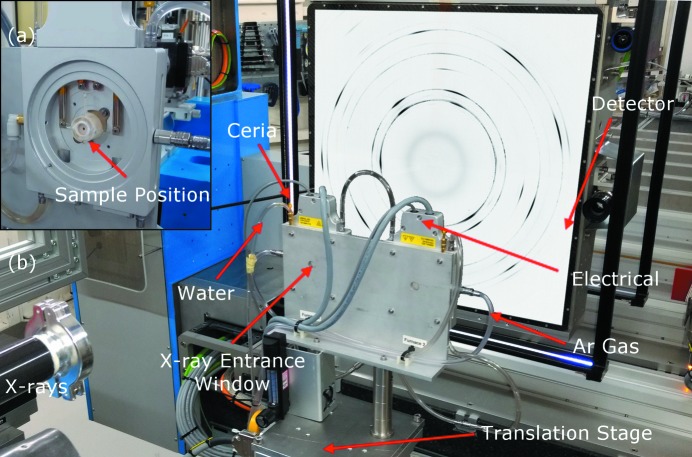
Image of the experimental configuration: (*a*) magnified view of a Linkam TS1500 furnace with exit window removed; (*b*) overview of the experimental apparatus in the I11 LDE hutch including a schematic two-dimensional powder diffraction pattern overlaid on the area detector.

Synchrotron X-ray powder diffraction (SXPD) measurements were performed on the new LDE facility, which is incorporated into beamline I11 at Diamond Light Source, UK. Two Linkam TS1500 stages were mounted on a heavy-duty goniometer. The experimental configuration and the stages are shown in Fig. 1 ▸, in which services such as water, gas and power needed to run the equipment are separately identified. The X-ray beam was monochromated to an energy of 25 keV (λ = 0.4959 Å) and had a beam size of 0.4 × 0.4 mm. A ceria powder standard (SRM674b) was mounted next to each stage, allowing calibration of the detector orientation angles, sample-to-detector distance and X-ray wavelength. Two-dimensional SXPD patterns were collected in transmission with a 60 s exposure using a Pixium RF4343 area detector at three different locations within the sample; these were averaged to produce a single powder diffraction pattern per data point.

The alloys then underwent a long duration thermal exposure; they were initially heated to 100°C at 10°C min^−1^ and held for 1 h to cure the small volume of alumina clay necessary to secure the sample within the crucible. The samples were then heated to 800°C and held at this temperature for the duration of the experiment. SXPD data were collected for the initial 15 h at a rate of 1 data point every 10 min. For the remaining exposure, the samples were measured at weekly intervals, for a total of 620 h for the 50Cr–25Co–25Ni and 50Cr–30Co–20Ni samples and 1170 h for the 50Cr–20Co–30Ni sample. In the interval between measurements, the Linkam stages remained in the beamline on a motorized stage, but placed in a parked condition so other experiments could proceed. Throughout the experiment, the samples were kept under an atmosphere of flowing argon to minimize the formation of oxides on their surfaces.

The two-dimensional diffraction patterns were azimuthally integrated using the software package *Nika* (Ilavsky, 2012 ▸), written as a plugin macro for the data analysis package *Wavemetrics Igor Pro *(https://www.wavemetrics.com) and using the instrumental parameters obtained from the calibration. Rietveld refinements of these data were performed using the diffraction data analysis software *TOPAS* (Coelho, 2018 ▸) to extract the lattice parameters (lp) and weight fractions (W_f_) of the phases.

Complementary microstructural characterization was performed on polished samples both before and after the long duration exposure. Backscattered electron imaging (BSEI) was completed using an FEI Nova NanoSEM 450 scanning electron microscope. Electron-backscattered diffraction (EBSD) and energy-dispersive X-ray spectroscopy (EDX) were completed on the same instrument using Bruker e^−^ flash^1000^ EBSD and Bruker XFlash 6 solid-state EDX detectors, respectively.

## 3. Results and discussion   

**Figure 2:**
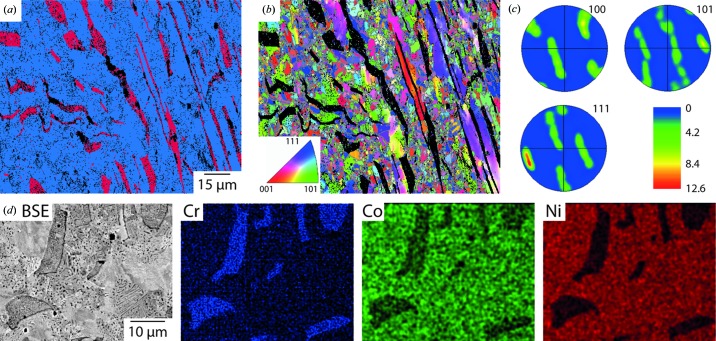
Microstructural characterization of the 50Cr–20Co–30Ni alloy prior to thermal exposure. (*a*) EBSD phase map. (*b*) Corresponding grain-orientation map. (*c*) Associated pole figures. (*d*) Higher-resolution BSEI image and accompanying EDX maps for the constituent elements.

The microstructure of the 50Cr–20Co–30Ni alloy following the annealing heat treatment is shown in Fig. 2 ▸. The EBSD phase map, Fig. 2 ▸(*a*), reveals a two-phase microstructure consisting of a gamma matrix (blue) and an alpha phase (A2, Struktubericht notation, red), along with regions of the sample that could not be successfully indexed using EBSD (black). Analysis of the gamma grain orientations and the associated pole figures, Figs. 2 ▸(*b*)–2(*c*), indicated that the annealing heat treatment had resulted in partial recrystallization of the alloy. The gamma phase, which constituted the majority of the microstructure, was composed of approximately 2–5 µm equiaxed grains in markedly different orientations interspersed with unrecrystallized regions that showed internal misorientations consistent with a high dislocation density from the rolling process. The corresponding pole figures, Fig. 2 ▸(*c*), showed significant texture that is likely a consequence of the unrecrystallized regions within the relatively small area studied. Complementary compositional analysis of the annealed microstructure is shown in Fig. 2 ▸(*d*). The BSEI image shows a dark phase within a lighter matrix. The accompanying EDX data are consistent with the lighter matrix being the gamma phase, which is principally an Ni–Co solid solution with a composition of 46Cr–21Co–33Ni, whilst the darker phase had a composition of 61Cr–18Co–21Ni. However, it should be noted that other fine-scale features could be identified in higher-magnification imaging of both regions that were beyond the resolution of the EDX technique, suggesting that both regions contained more than one phase.

**Figure 3:**
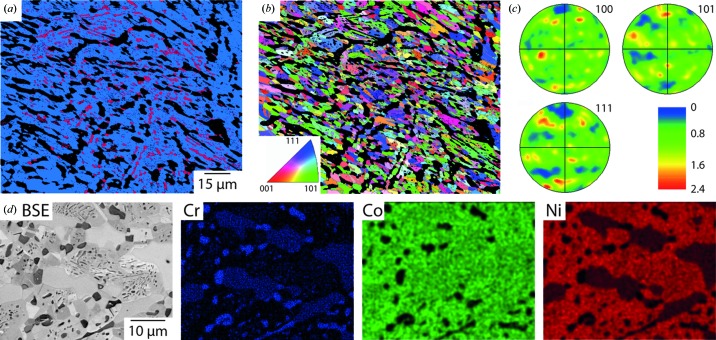
Microstructural characterization of the 50Cr–20Co–30Ni alloy after completion of the thermal exposure. (*a*) EBSD phase map. (*b*) Corresponding grain-orientation map. (*c*) Associated pole figures. (*d*) Higher-resolution BSEI image and accompanying EDX maps for the constituent elements.

On completion of the *in situ* thermal exposure, microstructural analyses were repeated and the results obtained from the 50Cr–20Co–30Ni alloy are presented in Fig. 3 ▸. The EBSD phase map, Fig. 3 ▸(*a*), shows a gamma matrix (blue), with a reduced fraction of the alpha phase (red) and an increased fraction of un-indexed regions (black). The analyses of the gamma grain orientations, Figs. 3 ▸(*b*)–3(*c*), revealed nearly equiaxed randomly orientated grains that were 5–10 µm in size, suggesting that the material had fully recrystallized. This is supported by the significantly lower maximum intensities associated with Fig. 3 ▸(*c*), which are around five times smaller than that in Fig. 2 ▸(*c*). The BSEI image and EDX maps presented in Fig. 3 ▸(*d*) revealed a change in the phase morphology as a result of the thermal exposure. Regions of the gamma matrix with the alpha phase were identified, along with substantial areas of a blocky intragranular phase. The Cr EDX map shows the underlying three-phase structure, with a very high Cr signal corresponding to the alpha phase present in both inter- and intra-granular positions, and a second slightly less Cr-rich phase as large regions adjacent to the gamma matrix. Related preferential elemental partitioning could also be observed in the Ni and Co EDX maps. Neither Co nor Ni showed much solubility in the Cr-rich alpha phase. However, significant Co levels were observed in the blocky intergranular phase, which were also depleted in Ni. Quantification of the EDX data revealed that the gamma phase had a composition of approximately 40Cr–23Co–37Ni and the alpha phase had a composition of approximately 93Cr–4Co–3Ni. The blocky intergranular phase was found to have a composition of approximately 64Cr–20Co–16Ni, which is consistent with known compositions of the sigma phase. Regions containing this phase were not indexed by EBSD due to difficulties in determining its structure from the Kikuchi patterns obtained. Similar microstructural observations were made on the 50Cr–25Co–25Ni and 50Cr–30Co–20Ni alloys, with the compositions of the sigma phases being approximately 61Cr–23Co–16Ni and 60Cr–26Co–14Ni, respectively. The persistence of the alpha phase after prolonged thermal exposure at 800°C is also notable as current published ternary phase diagrams are conflicted about the phases to be expected (Kaufman & Nesor, 1974 ▸; Zhmurko *et al.*, 2008 ▸).

**Figure 4:**
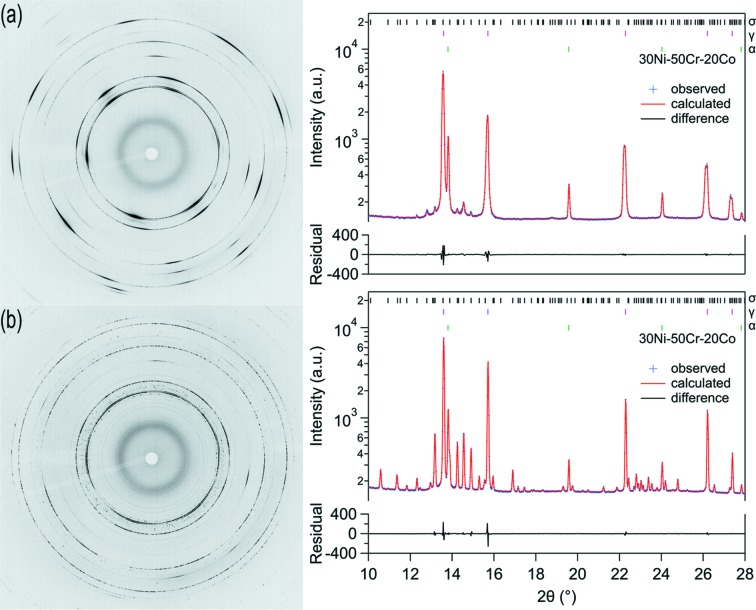
Synchrotron X-ray powder diffraction patterns from the 50Cr–20Co–30Ni alloy prior to thermal exposure (*a*) and after 1170 h at 800°C (*b*).

SXPD data collected from the 50Cr–20Co–30Ni alloy before and after thermal exposure are presented in Fig. 4 ▸. The figure includes the two-dimensional diffraction patterns and the azimuthally integrated data that have been Rietveld refined using the phases identified through microscopy. It should be noted that the diffuse ring observed at the smallest diffraction angle was associated with the mica windows of the high-temperature furnace used in the study and hence is not considered in the subsequent analyses.

The two-dimensional diffraction data obtained from the sample in the initial condition, Fig. 4 ▸(*a*), showed faint, continuous diffraction rings, with intensity modulations superimposed. These characteristics were most evident in the gamma phase, which was responsible for the strongest reflections in Fig. 4 ▸(*a*). The variations in diffracted intensity are consistent with the EBSD results discussed previously, Fig. 2 ▸(*b*), which identified larger grains with a similar orientation as well as more randomly orientated smaller grains.

Rietveld refinement of the azimuthally integrated data from the sample in the initial condition confirmed that the alloy comprised of three constituents, ∼88% gamma, ∼9% alpha and ∼3% sigma phase. Although the sigma phase was not directly identified in the microstructural analysis, it is believed that this small fraction of sigma phase is likely to be associated with the fine features that were not successfully indexed during the EBSD analysis.

**Figure 5:**
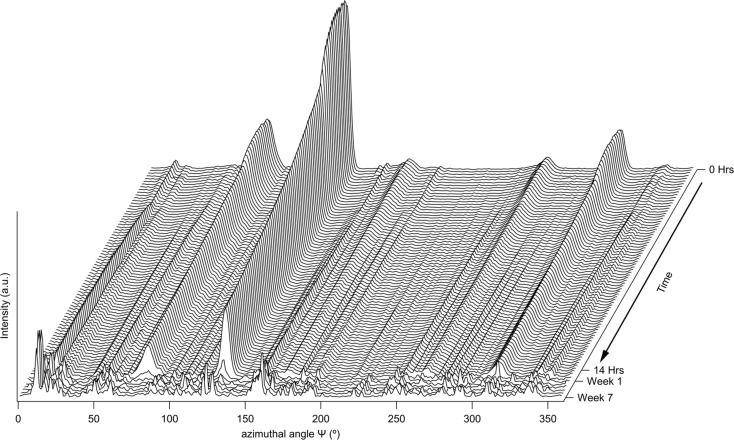
Waterfall plot of the variation of intensity of the {200} reflection from the gamma phase with azimuthal angle as a function of thermal exposure time.

Over the course of the initial 15 h of the experiment, changes were observed in the sample texture from the diffraction data. To illustrate these changes, a plot of the azimuthal variation in the intensity of the {200} reflection from the gamma phase as a function of exposure time is presented in Fig. 5 ▸. These data show progressive decreases in the localized intensity of this reflection at certain azimuthal angles, *e.g.* 80, 140 and 310°, as well as increasing intensity in other regions, *e.g.* between 210 and 230°. These observations indicated a continual recrystallization of the gamma phase, consistent with the microstructural observations made using EBSD on the initial and final states. After this initial 15 h data collection period, the next diffraction pattern was acquired one week later, by which time the strong texture that had originally been observed in the gamma phase had largely dissipated. All three of the alloys indicated similar textural evolutions during the course of their thermal exposures. Whilst it would have been desirable to monitor the textural and phase evolution with a higher data collection frequency throughout this period, the requirement to balance the needs of standard beamline access to the I11 high-resolution powder diffractometer with those of the I11 LDE facility limits individual LDE experiments to weekly diffraction data collection intervals after the initial setup period.

Throughout the experiment, the diffraction rings associated with the sigma phase were spotty, with no evidence of increased intensity at specific azimuthal angles. These observations were consistent with the formation of randomly orientated particles that were coarse compared with the diffraction gauge volume, as seen in previous studies (*e.g.* Liss *et al.*, 2006 ▸). The diffraction data obtained from the sample following thermal exposure, Fig. 4 ▸(*b*), showed that the fraction of sigma had increased significantly and both gamma and alpha phases were retained within the microstructure, entirely consistent with the microstructural observations.

**Figure 6:**
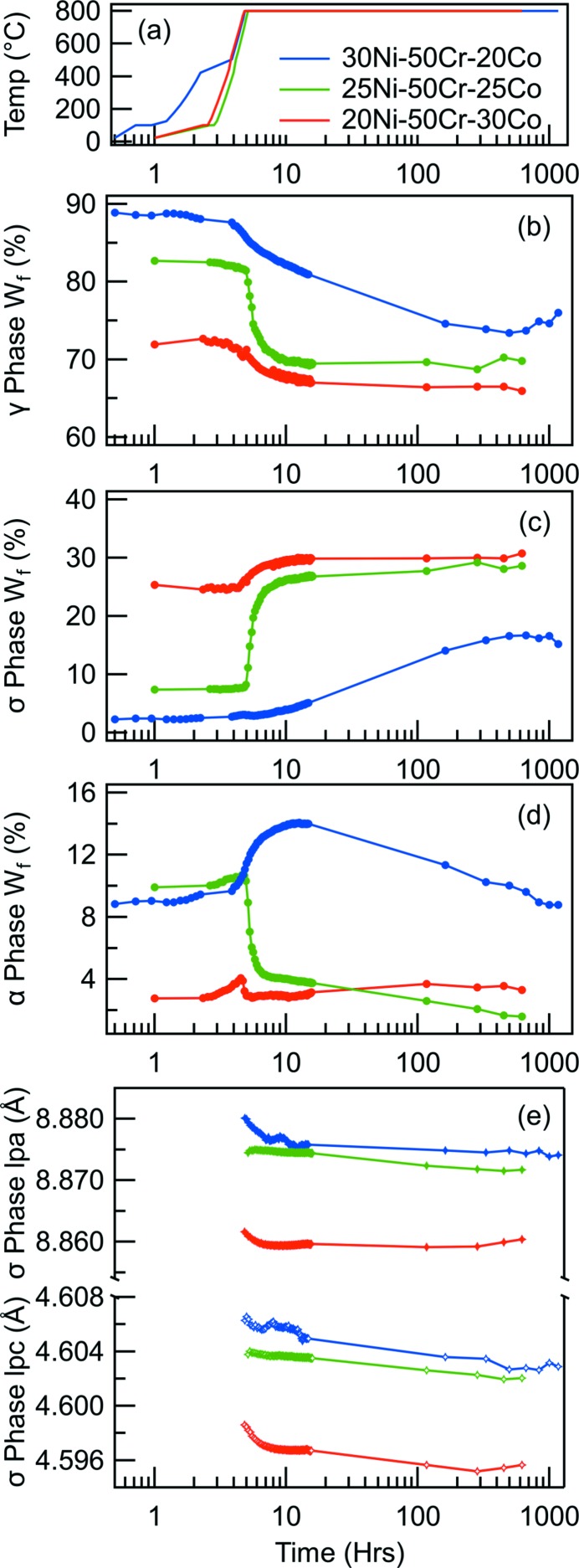
(*a*) Temperature profile of the three studied alloys. Temporal evolution of the (*b*) gamma, (*c*) sigma and (*d*) alpha phase weight fractions. (*e*) Lattice parameters of the sigma phase for the three alloys.

The temporal evolution of the volume fractions and lattice parameters of the phases present were obtained through Rietveld refinement of the diffraction patterns acquired at each time step, Fig. 6 ▸. Table 1 ▸ presents the crystallographic information used as the bases for each of the diffraction pattern refinements. Fig. 6 ▸(*a*) shows the thermal cycles experienced by each of the three alloys. The data for all of the alloys have been offset such that the start of the dwell at 800°C occurs at the 5 h mark. The fraction of the gamma, sigma and alpha phases in the alloys as a function of time are shown in Figs. 6(*b*), 6(*c*) and 6(*d*) ▸, respectively. In the initial condition the 50Cr–30Co–20Ni alloy contained the highest fraction of sigma ∼25%, whilst containing a small fraction of the alpha phase ∼3%. In contrast, the 50Cr–25Co–25Ni and 50Cr–20Co–30Ni alloys contained lower fractions of the sigma phase, ∼7% and ∼4%, respectively, as well as ∼9–10% of the alpha phase. This variation in the sigma and alpha fractions led to concomitant variations in the fraction of the gamma matrix, which varied from 88% to 72%.

During the heating ramps an increase in the alpha fraction was observed for all alloys, whilst the sigma fraction remained relatively unchanged. It should be noted that the 50Cr–20Co–30Ni alloy experienced a slower rate of heating, with an additional plateau at approximately 500°C. However, no significant changes to the volume fractions of any phases were observed as a consequence of the plateau.

The sigma phase fraction was observed to rise in all three alloys with increasing exposure time at 800°C. However, the rate at which this occurred and the evolution of the constituent phases differed markedly between the alloys. In the 50Cr–30Co–20Ni alloy, a significant fraction of sigma existed in the initial microstructure and it evolved quickly over the first 20 h at 800°C, with a comparatively small increase in the sigma fraction. This was accompanied by a similar decrease in the fraction of the gamma phase and a small decrease in that of the alpha phase. These observations suggest that sigma formation occurred in this alloy primarily at the expense of the gamma phase. The 50Cr–25Co–25Ni alloy showed similar phase evolution to the 50Cr–30Co–20Ni alloy, albeit the starting fraction of sigma was significantly lower and a larger increase in sigma phase fraction was observed over the first 20 h, such that it approached a value similar to the 50Cr–30Co–20Ni alloy. This was associated with marked decreases in the fractions of both the gamma and alpha phases, the latter of which continued to decrease throughout the duration of the thermal exposure to less than 2%. This suggests that the alpha phase may be metastable, as expected from published phase diagrams, and its presence may facilitate the formation of the sigma phase. In the 50Cr–20Co–30Ni alloy, sigma formation was observed to occur more sluggishly than the other two alloys, increasing in volume fraction over the course of the thermal exposure. Interestingly, the alpha phase fraction increased over the first 10 h before progressively decreasing over the remainder of the test. The initial increase in alpha phase fraction was accompanied by a decrease in the fraction of the gamma phase, suggesting that the alpha was drawing Cr out of the gamma. As the sigma phase began to form, the Cr was increasingly accommodated in the sigma phase, leading to a reduction in the alpha fraction. The changes in the phase fraction occurring up to 1000 h suggest that equilibrium may not have been reached and that further phase evolution may have occurred with longer duration exposure.

The variations in the lattice parameters of the sigma phase as a function of exposure time are shown in Fig. 6 ▸(*e*). The data acquired from all three alloys showed a progressive decrease in the lattice parameter with time and this was most marked in the initial hours of thermal exposure. These observations suggest that elemental redistribution between the phases had occurred during the thermal exposure or that inter-phase strain relaxation had occurred. However, it was not possible to decouple the relative contributions from these effects from the data obtained. In addition, the similarity between the lattice parameters of the sigma phase formed in the 50Cr–20Co–25Ni and the 50Cr–20Co–30Ni alloys suggest that the composition of these phases may be similar and different to that formed in the 50Cr–30Co–20Ni alloy. This may be attributable to distinct sigma phases occurring in these alloys, consistent with recent reports of the Cr–Co–Ni ternary system (Connor *et al.*, 2016 ▸). However, the EDX data from the sigma phases of the three alloys did not indicate a clear discontinuity in their compositions.

## 4. Conclusions   

Synchrotron diffraction data acquired during the *in situ* thermal exposure of three model Cr–Co–Ni ternary alloys has been used to demonstrate the capabilities of the new long duration experiment (LDE) facility at Diamond Light Source. All three alloys contained gamma, sigma and alpha phases in the initial condition, the fraction of which varied with alloy composition. During thermal exposure the sigma phase fraction increased in all three alloys, although the evolution of other phases showed distinct differences, particularly in the fraction of the alpha phase present. These observations were rationalized through elemental redistribution during thermal exposure. In addition, the textural evolution in the samples was characterized and shown to correlate with EBSD data.

The results obtained using the LDE facility have provided new insights into the temporal evolution of phases during thermal exposure of alloys that could not have been readily achieved using *ex situ* experiments and over a duration that cannot be routinely accessed at synchrotron radiation facilities. In this regard, this work demonstrates, for the first time, the unique capabilities of the LDE facility on beamline I11 at Diamond Light Source, which offers the combination of high X-ray fluxes and high-quality diffraction data for experimental studies lasting months to years. Whilst this study has used a metallurgical test case to demonstrate these capabilities, the use of the LDE facility may be extended to almost any system that involves crystallographic changes occurring over long time scales. It therefore has potential applicability to a very wide range of research fields from textural evolution in geological systems to crystallographic changes in pharmaceuticals.

## Figures and Tables

**Table 1:** Crystallographic phase data

Crystal data	γ	σ	α
Phase chemistry	Cr_0.5_Co_0.3_Ni_0.2_	Cr_0.65_Co_0.2_Ni_0.15_	Cr
System	Cubic	280	Cubic
Space group	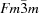	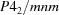	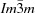
*a*, *b*, *c* (Å)	3.614	8.874, 8.874, 4.603	2.905
